# SARS-CoV-2 Omicron variant causes brain infection with lymphoid depletion in a mouse COVID-19 model

**DOI:** 10.1186/s42826-023-00157-4

**Published:** 2023-05-09

**Authors:** Na Yun Lee, Youn Woo Lee, Seung-Min Hong, Dain On, Gyeong Min Yoon, See-He An, Ki Taek Nam, Jun-Young Seo, Jeon-Soo Shin, Yang-Kyu Choi, Seung Hyun Oh, Jun-Won Yun, Ho Young Lee, Kang-Seuk Choi, Je Kyung Seong, Jun Won Park

**Affiliations:** 1 Division of Biomedical Convergence, College of Biomedical Science, Kangwon National University, 1 Kangwondaehak-Gil, Chuncheon-si, Gangwon-do 24341 South Korea; 2 Department of Nuclear Medicine, Seoul National University Bundang Hospital, Seoul, South Korea; 3 Laboratory of Avian Diseases, BK21 Plus Program for Veterinary Science and Research Institute for Veterinary Science, College of Veterinary Medicine, Seoul National University, Seoul, South Korea; 4 Korea Mouse Phenotyping Center (KMPC), Seoul National University, Seoul, 08826 South Korea; 5 Laboratory of Developmental Biology and Genomics, Research Institute for Veterinary Science, and BK 21 PLUS Program for Creative Veterinary Science Research, College of Veterinary Medicine, Seoul National University, Seoul, 08826 South Korea; 6 Avian Influenza Research and Diagnostic Division, Animal and Plant Quarantine Agency, Gimcheon, 39660 South Korea; 7 Severance Biomedical Science Institute, Brain Kore a 21 FOUR Project for Medical Science, Yonsei University College of Medicine, Seoul, 03722 South Korea; 8 Institute of Immunology and Immunological Diseases, Yonsei University College of Medicine, Seoul, 03722 South Korea; 9 Department of Microbiology, Yonsei University College of Medicine, Seoul, 03722 South Korea; 10 Department of Laboratory Animal Medicine, College of Veterinary Medicine, Konkuk University, Seoul, 05029 South Korea; 11 College of Pharmacy, Gachon University, Incheon, South Korea; 12 Laboratory of Veterinary Toxicology, College of Veterinary Medicine, Seoul National University, Seoul, 08826 South Korea

**Keywords:** Animal model, K18-hACE2, Lymphoid depletion, Wuhan

## Abstract

**Background:**

The Omicron variant has become the most prevalent SARS-CoV-2 variant. Omicron is known to induce milder lesions compared to the original Wuhan strain. Fatal infection of the Wuhan strain into the brain has been well documented in COVID-19 mouse models and human COVID-19 cases, but apparent infections into the brain by Omicron have not been reported in human adult cases or animal models. In this study, we investigated whether Omicron could spread to the brain using K18-hACE2 mice susceptible to SARS-CoV-2 infection.

**Results:**

K18-hACE2 mice were intranasally infected with 1 × 10^5^ PFU of the original Wuhan strain and the Omicron variant of SARS-CoV-2. A follow-up was conducted 7 days post infection. All Wuhan-infected mice showed > 20% body weight loss, defined as the lethal condition, whereas two out of five Omicron-infected mice (40%) lost > 20% body weight. Histopathological analysis based on H&E staining revealed inflammatory responses in the brains of these two Omicron-infected mice. Immunostaining analysis of viral nucleocapsid protein revealed severe infection of neuron cells in the brains of these two Omicron-infected mice. Lymphoid depletion and apoptosis were observed in the spleen of Omicron-infected mice with brain infection.

**Conclusion:**

Lethal conditions, such as severe body weight loss and encephalopathy, can occur in Omicron-infected K18-hACE2 mice. Our study reports, for the first time, that Omicron can induce brain infection with lymphoid depletion in the mouse COVID-19 model.

**Supplementary Information:**

The online version contains supplementary material available at 10.1186/s42826-023-00157-4.

## Background

Coronavirus disease 2019 (COVID-19) is a contagious disease caused by a respiratory infection of severe acute respiratory syndrome coronavirus 2 (SARS-CoV-2). Several variants of SARS-CoV-2 have emerged since the end of 2019, beginning with the original Wuhan strain, followed by the Beta, Delta, and Omicron variants [1]. Currently, the Omicron variant has become the dominant strain, resulting in a pattern of development into the prolonged and endemic COVID-19 [2].

Previous studies have shown that different strains of SARS-CoV-2 have different symptom profiles. The Omicron variant is known to induce relatively few pulmonary lesions, although it is much more contagious than previous strains [3]. The Omicron variant results in relatively lower viral titers and milder pneumonia in the lungs compared to the original Wuhan strain or the Delta variant in mouse and hamster COVID-19 models [4]. Notably, while the original strain caused fatal brain infections in COVID-19 mouse models [5, 6], apparent brain infections reportedly did not manifest in Omicron-infected animal models as well as adult human cases. However, the severity of SARS-CoV-2 variants is multifaceted because of varying levels of individual immune status, which calls for caution against underestimating the pathogenicity induced by the Omicron variant. Recently, severe encephalopathy associated with the Omicron BA.1 variant was reported in a neonate [7].

The human angiotensin-converting enzyme 2 (hACE2) protein is the functional receptor required for SARS-CoV-2 to enter cells [8]. Transgenic mice expressing hACE2 under the cytokeratin 18 promoter (K18-hACE2) represent a lethal model of SARS-CoV-2 infection [9]. Here, we aimed to demonstrate the occurrence of brain infection caused by the Omicron variant using K18-hACE mice. We found that two out of five Omicron-infected mice had brain infections. The clinicopathological parameters and immunological characteristics of Omicron-infected mice with brain infection were investigated.

## Materials and methods

### Virus production and titration

The original Wuhan strain of SARS-CoV-2 (accession number: NCCP43326/Korea) and the SARS-CoV-2 Omicron variant (BA.1, accession number: NCCP43408/Korea) were obtained from the Korea Centers for Disease Control and Prevention. SARS-CoV-2 was inoculated with Vero E6 cells (CRL-1586) to confirm the cytopathic effect on the third day. Virus titer was measured by plaque assay.

The tissue culture infectious dose endpoint (TCID50) was calculated according to the Reed–Muench method [10]. The Vero E6 cells were seeded in 12-well plates at a concentration of 3 × 10^5^ cells per well and incubated to form a monolayer one day prior to the plaque assay. The cells were infected for 1 h in duplicate with 10-fold serial dilutions of SARS-CoV-2 and overlaid with a 0.3% SeaPlaque (LONZA, Basel, Switzerland) agarose medium containing 2% FBS. After 72 h of incubation, the cells infected with the viruses were fixed using 4% (v/v) paraformaldehyde (Biosesang, Seongnam, Korea) for 1 h and then stained using a crystal violet solution (548-62-9, Sigma–Aldrich, St. Louis, United States). The infectious virus titers were measured in plaque-forming units (PFU) per ml.

### Animal model

The K18-hACE2 mice (8 weeks old, male) used in these studies were purchased from the Jackson Laboratory (B6. Cg-Tg(K18-*ACE2*)2Prlman/J). All protocols were approved by the Institutional Animal Care and Use Committee of the Seoul National University Bundang Hospital (IACUC number BA-2008-301-071-05). All animals were cared for in accordance with the Institute for Laboratory Animal Research Guide for the Care and Use of Laboratory Animals Eighth Edition [11]. The Seoul National University Bundang Hospital Institutional Biosafety Committee (IBC-2109-A-001) approved the procedures for sample handling, inactivation, and transfer from animal biosafety level 3 (ABSL3) containment.

All mice were infected with viruses intranasally with a total volume of 50 μl DMEM. The mice were lightly anesthetized with a mixture of ketamine (20 mg/kg) and xylazine (10 mg/kg). The weight, temperature, and health of the mice were monitored daily.

### Histopathology and immunostaining

Lung, spleen, and brain tissues were fixed in neutral buffered 10% formalin for 1 day and processed using a standard method. The 3-μm paraffin-embedded sections were stained with hematoxylin and eosin (H&E) for histopathological analyses. The lesions were graded using a semiquantitative scale based on the percentage of tissue affected by pathological changes as follows: 0, absent; 1, minimal, fewer than 10% of tissue affected; 2, mild, more than 10% but less than 25% of tissue affected; 3, moderate, more than 25% but less than 50% of tissue affected; 4, moderately severe, more than 50% but less than 75% of tissue affected; or 5, severe, more than 75% of tissue affected.

For immunohistochemistry (IHC), paraffin sections were dewaxed, rehydrated, and subjected to antigen retrieval by heating at 100 °C for 20 min in 0.01 M citrate buffer (pH 6.0) (C9999; Sigma-Aldrich). The ImmPRESS Peroxidase Polymer kit (Vector Laboratories, Burlingame, United States) was used in accordance with the manufacturer's protocol for immunostaining. Rabbit anti-SARS-CoV-2 nucleocapsid antibodies (Genetex, Irvine, United States) were used as the primary antibodies. The slides were subjected to colorimetric detection with the ImmPact DAB substrate (SK-4105; Vector Laboratories). The slides were counterstained with Meyer's hematoxylin for 10 s. Negative controls were performed by omitting the primary antibody and substituting it with diluent.

For immunofluorescence (IF) staining, formalin-fixed paraffin-embedded (FFPE) slides were deparaffinized and sequentially rehydrated using ethanol. The slides were immersed in antigen retrieval solution (DAKO, Santa Clara, United States) and incubated for antigen retrieval at high pressure using a cooker. After cooking, the slides were incubated with Antibody Diluent with Background Reducing Components (DAKO) for blocking and then incubated overnight at 4 °C with the primary antibodies. Mouse anti-SARS-CoV-2 nucleocapsid (Sino Biological), rabbit anti-SARS-CoV-2 nucleocapsid (Genetex, Irvine, United States), rabbit anti-CD19 (Cell Signaling Technology), rat anti-CD3 (Abcam), and mouse anti-neuron-specific enolase (DAKO, Santa Clara, United States) antibodies were used as primary antibodies. The primary antibodies were derived from diverse species, and each was detected using Alexa488-, and Alexa568-conjugated secondary antibodies (Invitrogen, Waltham, United States). Nuclear staining was performed using 4′,6-Diamidino-2-phenylindole dihydrochloride (DAPI) (Abbkine, Wuhan, China). Confocal images were taken with a confocal microscope (Ts2, Nikon, Japan) at the Kangwon Center for System Imaging (KCSI).

### TUNEL assay

The detection of apoptotic cells in tissue sections was performed using the terminal deoxynucleotidyl transferase dUTP nick end labeling (TUNEL) assay and the in situ Apoptosis Detection Kit (MK500; Takara Biotechnology, Dalian, China), in accordance to the manufacturer’s instructions.

### In situ hybridization (ISH)

SARS-CoV-2 spike protein mRNA was detected by in situ hybridization (ISH) using RNAscope Probes (ACD, Bio-Techne, MN, USA) and the RNAscope® 2.5 HD Duplex Assay (322436; ACD, Bio-Techne). Briefly, paraffin sections were retrieved in a boiling buffer and treated with protease for 30 min. In situ hybridization was performed according to the manufacturer’s protocol.

### Statistical analyses

Statistical analyses were performed with the GraphPad Prism version 8 software. Error bars indicate the standard deviations (SDs) from the mean. Significance is indicated as follows: *p < 0.05, **p < 0.01, and ***p < 0.001.

## Main text

### The Omicron variant shows milder clinical symptoms than the original Wuhan strain, but there are greater individual differences in severity.

**Fig. 1:**
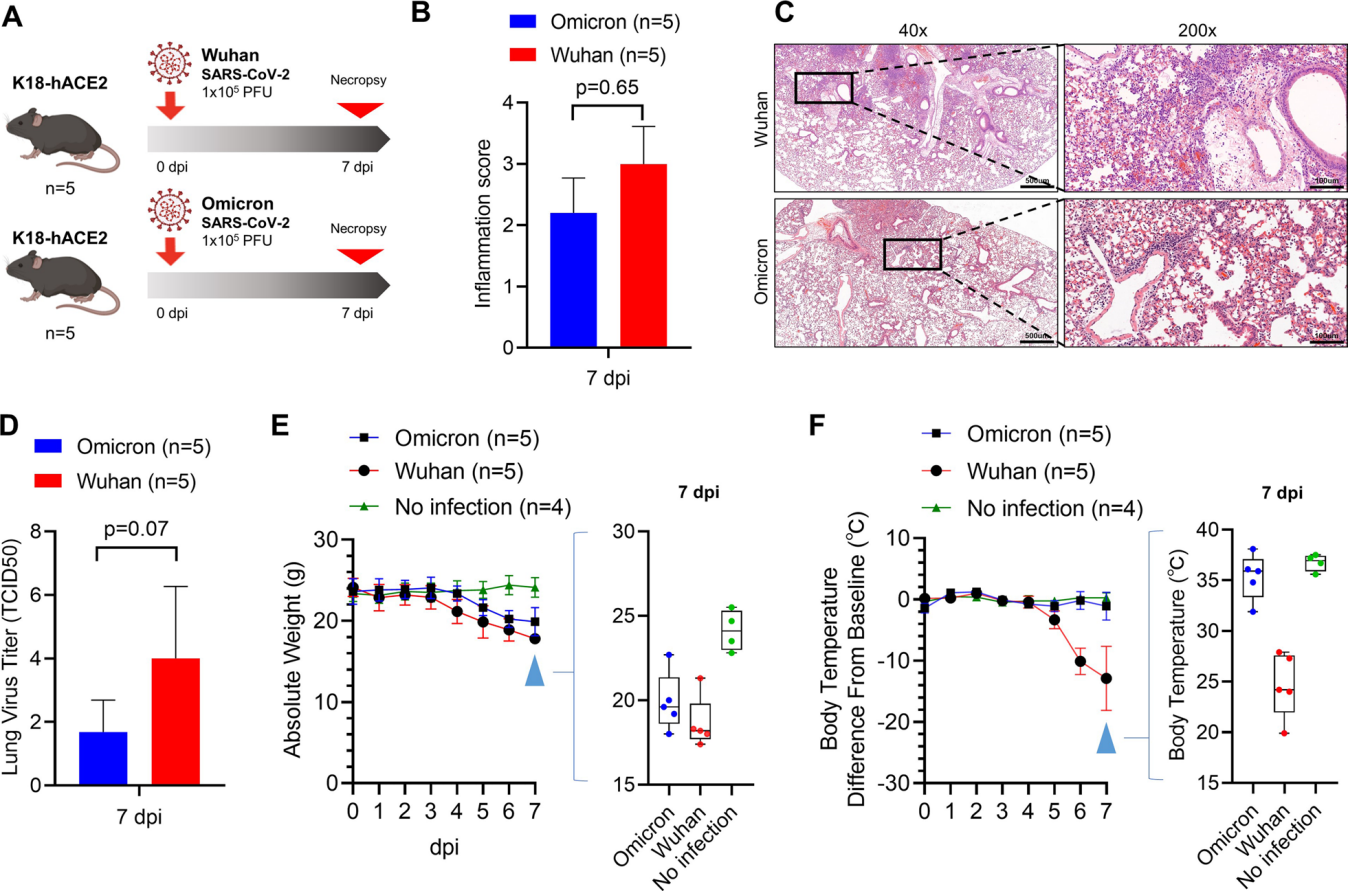
Comparison of clinicopathological characteristics of Wuhan- and Omicron-infected K18-hACE2 mice. **A** K18-hACE2-transgenic mice inoculated with 1 × 10^5^ PFU Wuhan and Omicron SARS-CoV-2 via the intranasal route. They were necropsied at 7 dpi. **B** The histopathological score of lung inflammation at 7 dpi was measured based on H&E slides. **C** Representative images of H&E staining of virus-infected lung tissues at 7 dpi. Boxed areas are magnified in the right panel. Scale bar = 500 μm (40×) and 100 μm (200×). **D** Viral burden in the lungs was measured at 7 dpi by TCID50. **E** The weight of the mice was monitored. Right graph, the individual values of the mice at 7 dpi. **F** The body temperature of the mice was monitored. Body temperature was presented with the difference from baseline. Baseline, the average body temperature of uninfected normal mice. Right graph, individual values of the mice at 7 dpi. Student’s-t test, *p < 0.05; **p < 0.01; ***p < 0.001. Error bars indicate the mean ± SD

The K18-hACE2 mice were inoculated through the intranasal route with 10^5^ PFU of the Omicron variant and the Wuhan strain (Fig. 1A). A decreasing trend in lung inflammation and virus titer was observed in mice infected with the Omicron variant compared to the Wuhan strain at 7 days post infection (dpi) (Figs. 1B, C and D). Mice infected with the Wuhan strain showed > 20% weight loss and > 10 °C temperature drop on average at 7 dpi, reaching the moribund state (Figs. 1E, F). Although the weight loss and body temperature drops of Omicron-infected mice were lower than those of Wuhan-infected mice (Figs. 1E, F), a wider range of individual differences in the weight loss was observed. The weight loss of Wuhan-infected mice ranged from − 17.2 to − 26.2% (SD = 3.6%; Fig. 1E), whereas that of Omicron-infected mice ranged from − 2.2 to − 25.8 (SD = 9.0%; Fig. 1E). In two of five Omicron-infected mice, fatal weight loss of > 20% also occurred (Fig. 1E). This result revealed that the Omicron variant induced milder clinical symptoms compared to the Wuhan strain and that individual differences with regard to severity were relatively greater.

### Viral infection into the brain of Omicron-infected mice with clinical severity

**Fig. 2:**
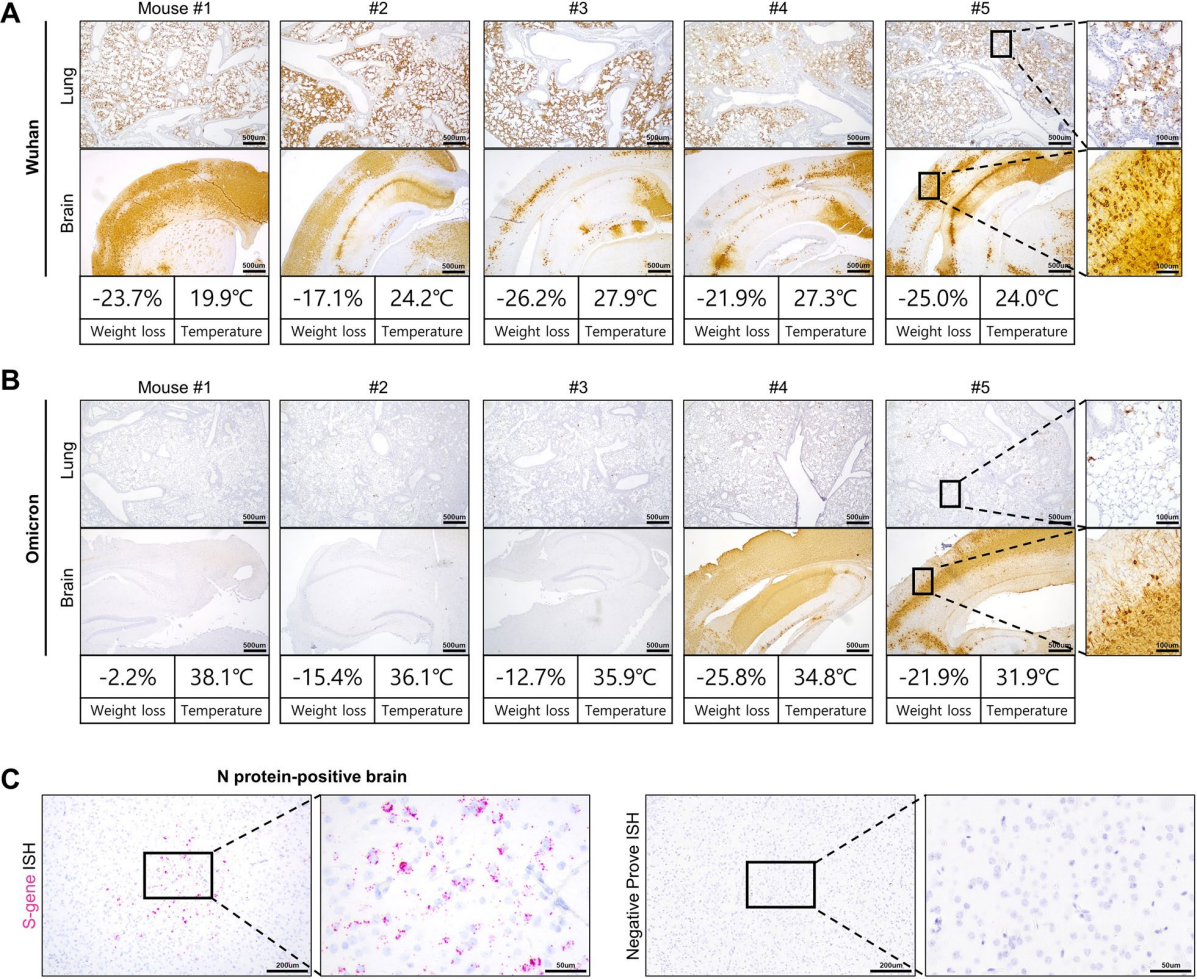
Comparison of viral infection in the lungs and brains of Wuhan- and Omicron-infected K18-hACE2 mice. **A** IHC images for SARS-CoV-2 N protein in the lungs and brains of Wuhan-infected K18-hACE2 mice at 7 dpi. Scale bar = 500 μm (low power) and 100 μm (high power). The body weight and temperature of each mouse are provided below the IHC images. **B** IHC images of SARS-CoV-2 N protein in the lungs and brains of Omicron-infected K18-hACE2 mice at 7 dpi. Scale bar = 500 μm (low power) and 100 μm (high power). The body weight and temperature of each mouse are provided below the IHC images. **C** Representative ISH images of viral spike protein mRNA (S gene, Red) in the brain of Omicron-infected mouse #5. Scale bar = 200 μm (low power) and 50 μm (high power). The boxed areas are magnified in the right panels

The viral infection and distribution in the lungs and brains of the mice were investigated through IHC analysis for viral nucleocapsid (N) protein. The specificity of the antibody used in this study was verified by confirming that two different antibodies showed the same IF stain patterns (Additional file 1: Figure S1). In the lungs of Wuhan-infected mice, N protein was widely detected not only in inflammatory areas but also in non-inflamed ones (Fig. 2A). N protein was extensively detected in the brain tissues, including the cerebral cortexes, of all Wuhan-infected mice (Fig. 2A).

**Fig. 3:**
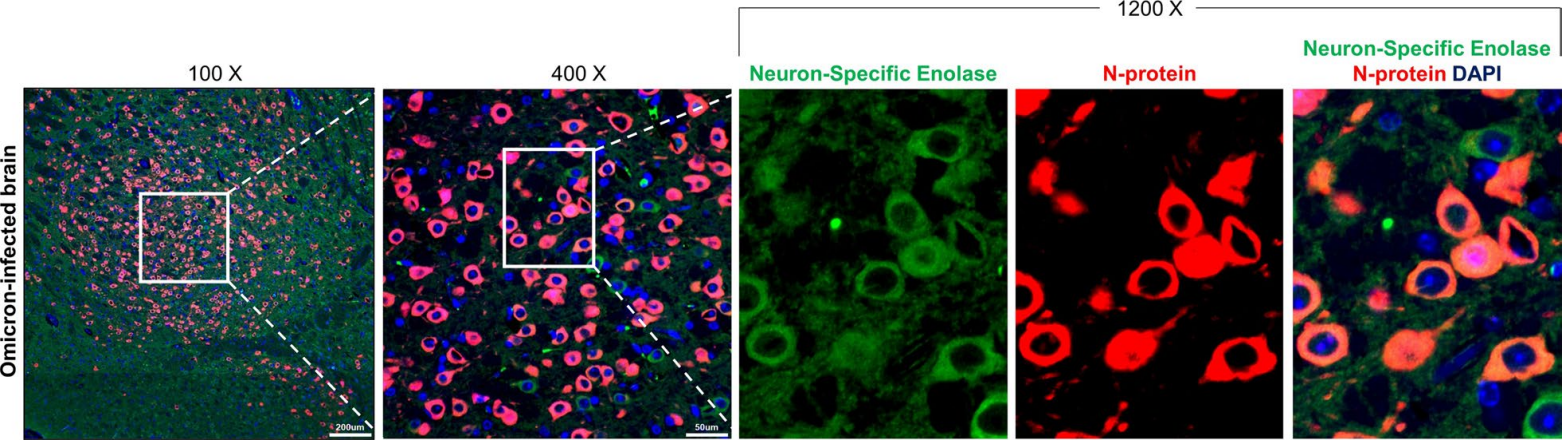
Nerve cell infection in the brain of an Omicron-infected mouse. Representative IF images for neuron-specific enolase (green; a neuron marker) and viral N protein (red) in the brains of Omicron-infected K18-hACE2 mice with brain infection at 7 dpi. Boxed areas are magnified in the right panels. Scale bar = 200 μm (100×) and 50 μm (400×)

In the lungs of the Omicron-infected mice, N protein was multifocally detected in a much smaller area than in the lungs of the Wuhan-infected mice (Fig. 2B). Based on H&E analysis, the presence of perivascular cuffing by mononuclear cells was observed (Additional file 2: Figure S2), which provides evidence of viral invasion into the brain. Notably, IHC analysis of N protein revealed that two Omicron-infected mice showed severe brain infection (Fig. 2B). Brain infection was also verified using ISH analysis of viral spike protein mRNA (Fig. 2C). The Omicron variant mainly infected brain nerve cells, based on IF analysis using a nerve cell marker and viral N protein (Fig. 3). These two mice showed greater weight loss and body temperature drops compared to the other Omicron-infected mice (Fig. 2B). This result indicates that brain infection with the Omicron variant can occur in mice with clinical severity.

### Lymphoid depletion in Omicron-infected mice with brain infection

**Fig. 4:**
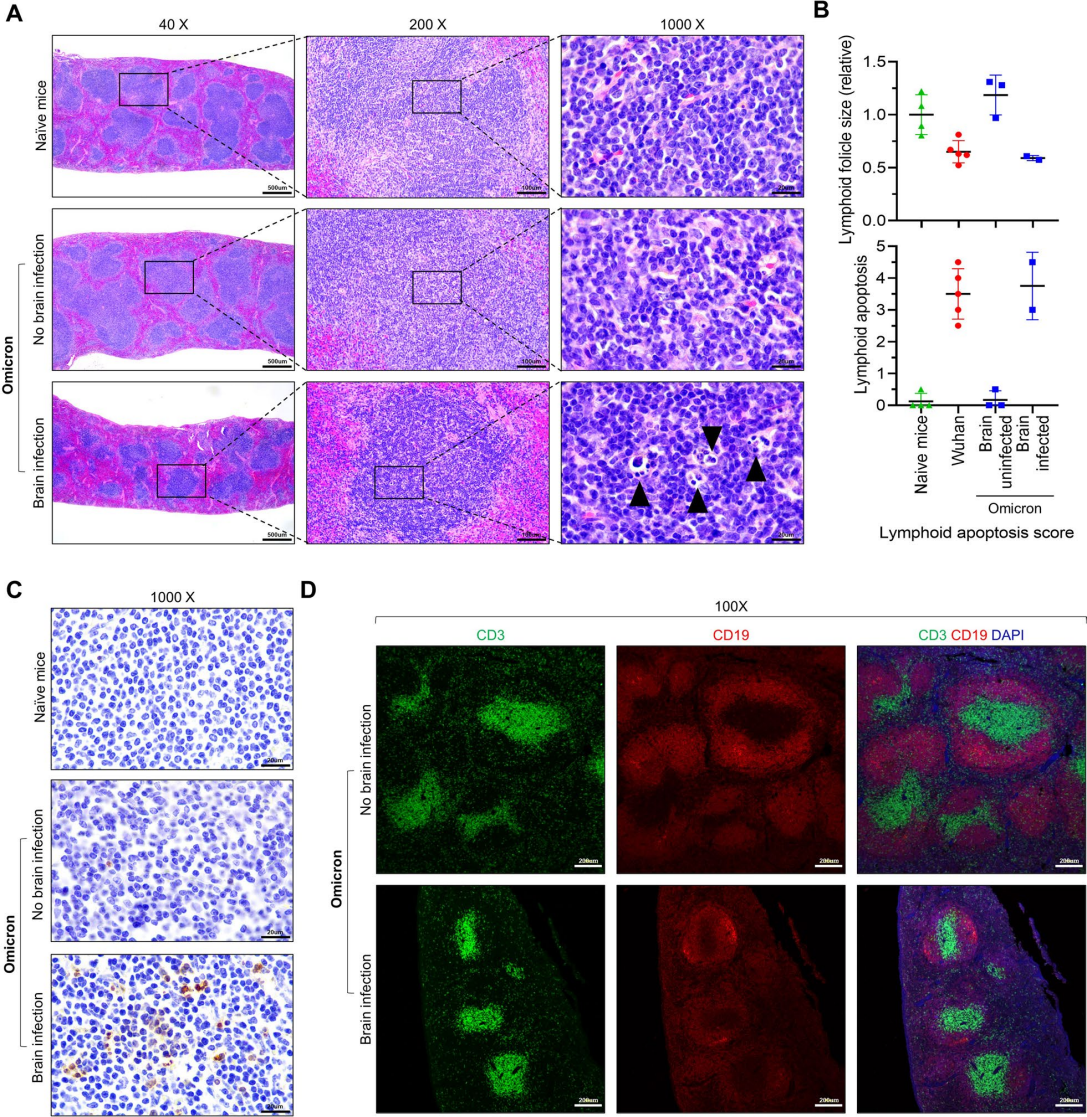
Lymphoid depletion in Omicron-infected mice with brain infection **A** Representative H&E images of the spleens from Omicron-infected K18-hACE2 mice with brain infection at 7 dpi. Arrow heads, apoptotic cells. **B** Histological scores of lymphoid depletion and apoptosis in white pulp of the spleen based on H&E slides. **C** Representative TUNEL images of the spleens from Omicron-infected K18-hACE2 mice with brain infection at 7 dpi. **D** Representative IF images of CD3 + T cells and CD19 + B cells in the spleen of an Omicron-infected K18-hACE2 mouse with brain infection at 7 dpi. CD3 (green), CD19 (red), and DAPI (blue; nucleus). Boxed areas were magnified in the right panel. Scale bar = 500 μm (40×), 200 μm (100×), 100 μm (200×), and 20 μm (1000×)

The risk of spread of SARS-CoV-2 throughout the body, including the brain, may be associated with dysregulated immunity in COVID-19 patients [12]. The spleen is a major lymphoid organ that captures blood borne antigens and can reflect systemic immune status. Notably, based on H&E analyses, severe atrophy of the white pulp in the spleen of Omicron-infected mice with brain infection was found (Fig. 4A, B). Apoptotic cells characterized by nuclear pyknosis, karyolysis, and karyorrhexis were frequently detected in the white pulp region of the brain-infected mice (Fig. 4A, B). TUNEL assay additionally confirmed the detection of white pulp apoptosis (Fig. 4C). Using double IF staining for CD3 and CD19 (Fig. 4D), atrophy of the white pulp was characterized by T- and B-lymphocyte depletion. These results indicate that Omicron-infected mice with brain infections also showed abnormalities in adaptive immunity.

## Conclusions

The clinical significance of brain infection with SARS-CoV-2 is a topic of active research. COVID-19 patients with neurological symptoms, including headache, dizziness, myalgia, confusion, delirium, and altered mental status have been found to be associated with brain infection and inflammation [13, 14]. Some studies have suggested that Central Nervous System (CNS) involvement in COVID-19 may be associated with more severe disease and a higher risk of death [12]. In this study, we revealed that the Omicron variant can infect the brain in K18-hACE2 mice. Similar to our observations, it has been reported that the Omicron virus causes attenuated lung lesions in animal models [4]. However, brain infection caused by the Omicron virus has not been reported in COVID-19 animal models or in adult human cases. Importantly, this is the first study to report apparent brain infection with the Omicron virus in an animal model. Our results also showed that Omicron infection in the brain is accompanied by abnormalities in lymphoid tissues. This study calls for caution against underestimating the pathogenicity induced by the Omicron variant. In certain situations, especially in patients with immune disorders due to underlying diseases, Omicron variant infection may cause sequelae related to infection in the brain.

## Supplementary Information

**Additional file 1**. **Figure 1.** Representative IF images for viral nucleocapsid using antibodies from different hosts in the brain of a SARS-CoV-2-infected K18-hACE2 mouse with brain infection. Scale bar = 50 μm (400×).

**Additional file 1**. **Figure 2.** Representative H&E images for perivascular cuffing in the brains of Omicron-infected K18-hACE2 mice with brain infection. Scale bar = 50 μm (400×).

## Data Availability

The datasets used and/or analyzed during the current study are available from the corresponding author on reasonable request.

## Abbreviations

ACE2: Angiotensin-converting enzyme 2
COVID-19: Coronavirus disease 2019
DPI: Days post infection
K18: Cytokeratin 18
SARS-CoV-2: Severe acute respiratory syndrome coronavirus 2
