# Supplementary figures and images for: SARS-CoV-2 Omicron variant causes brain infection with lymphoid depletion in a mouse COVID-19 model
Source: Lab Anim Res. 2023 May 9;39:8. doi: 10.1186/s42826-023-00157-4 (PMC10169124; doi:10.1186/s42826-023-00157-4)

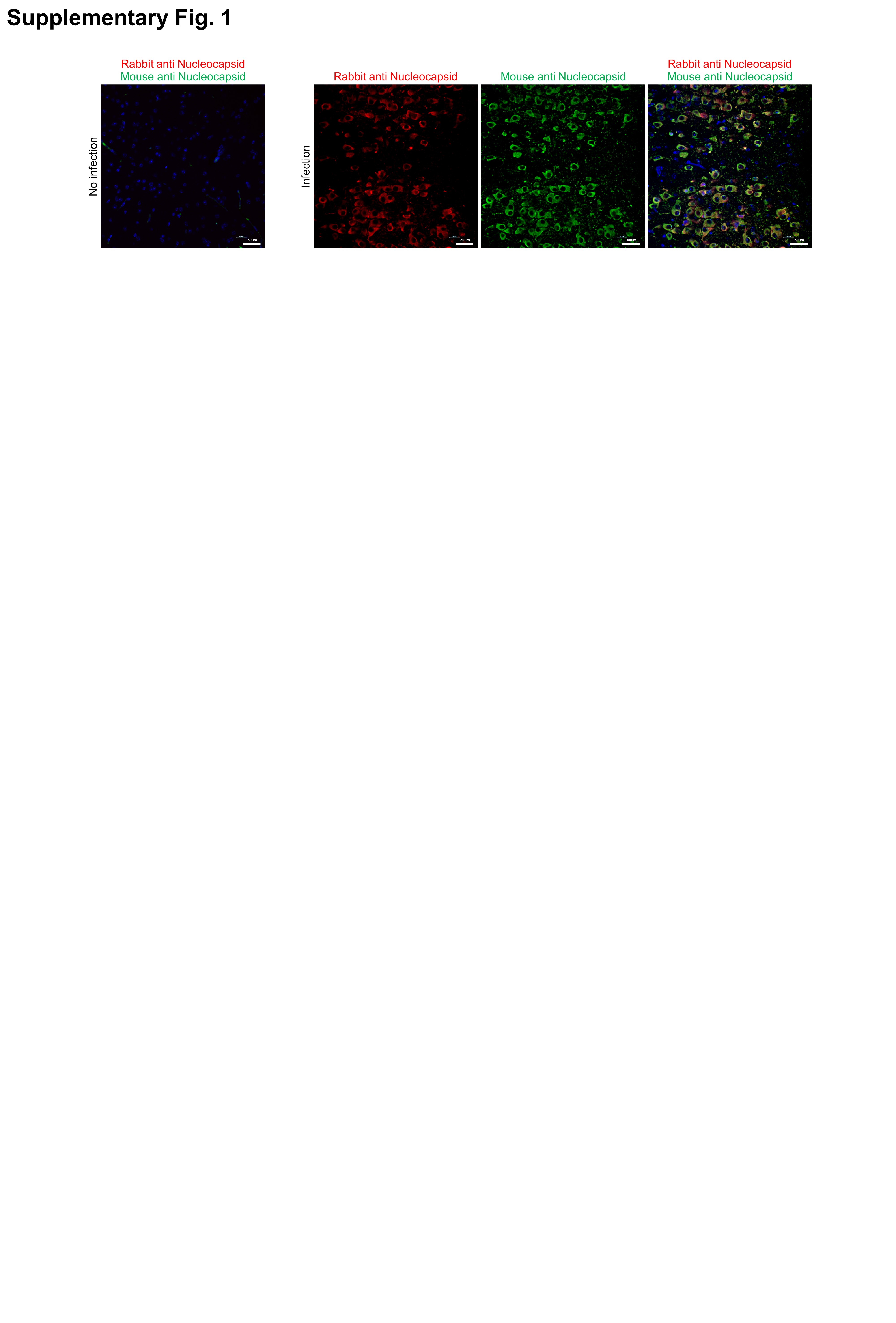

Supplement: Supplementary file 1 — Additional file 1. Figure 1. Representative IF images for viral nucleocapsid using antibodies from different hosts in the brain of a SARS-CoV-2-infected K18-hACE2 mouse with brain infection. Scale bar = 50 μm (400×). [file 42826_2023_157_MOESM1_ESM.jpg]

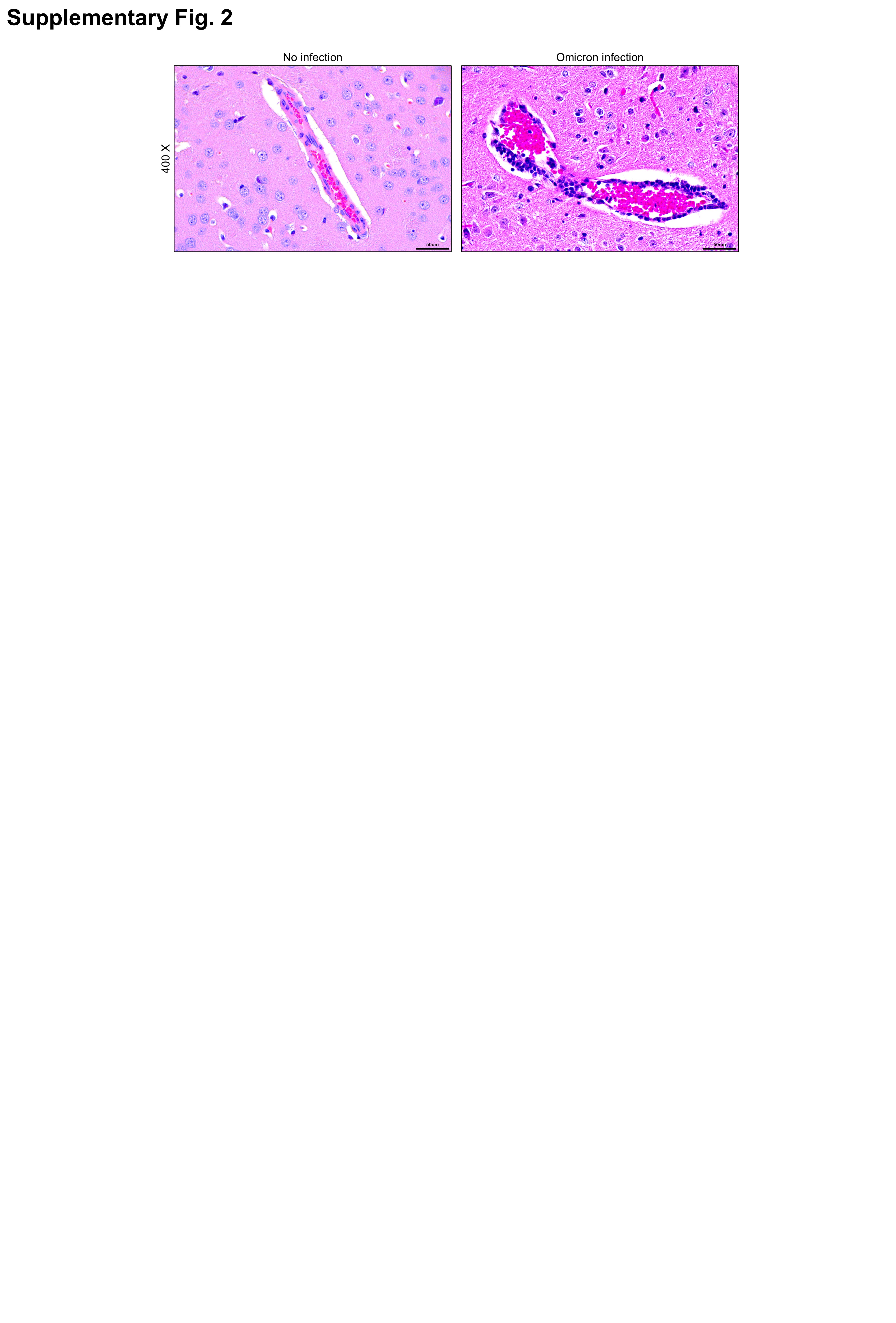

Supplement: Supplementary file 2 — Additional file 1. Figure 2. Representative H&E images for perivascular cuffing in the brains of Omicron-infected K18-hACE2 mice with brain infection. Scale bar = 50 μm (400×). [file 42826_2023_157_MOESM2_ESM.jpg]
